# Supplementary material for: Apple Ripening Is Controlled by a NAC Transcription Factor
Source: Front Genet. 2021 Jun 22;12:671300. doi: 10.3389/fgene.2021.671300 (PMC8258254; doi:10.3389/fgene.2021.671300)
Supplement: Supplementary Figure 1 — Correlations among phenotypes. The distributions of each phenotype are shown as well as dot plots of comparisons between each pair of phenotypes. The results of a Pearson correlation test are provided for each pairwise comparison. [file Data_Sheet_1.zip › Supplementary files/Supp Figures/Image 1.pdf]

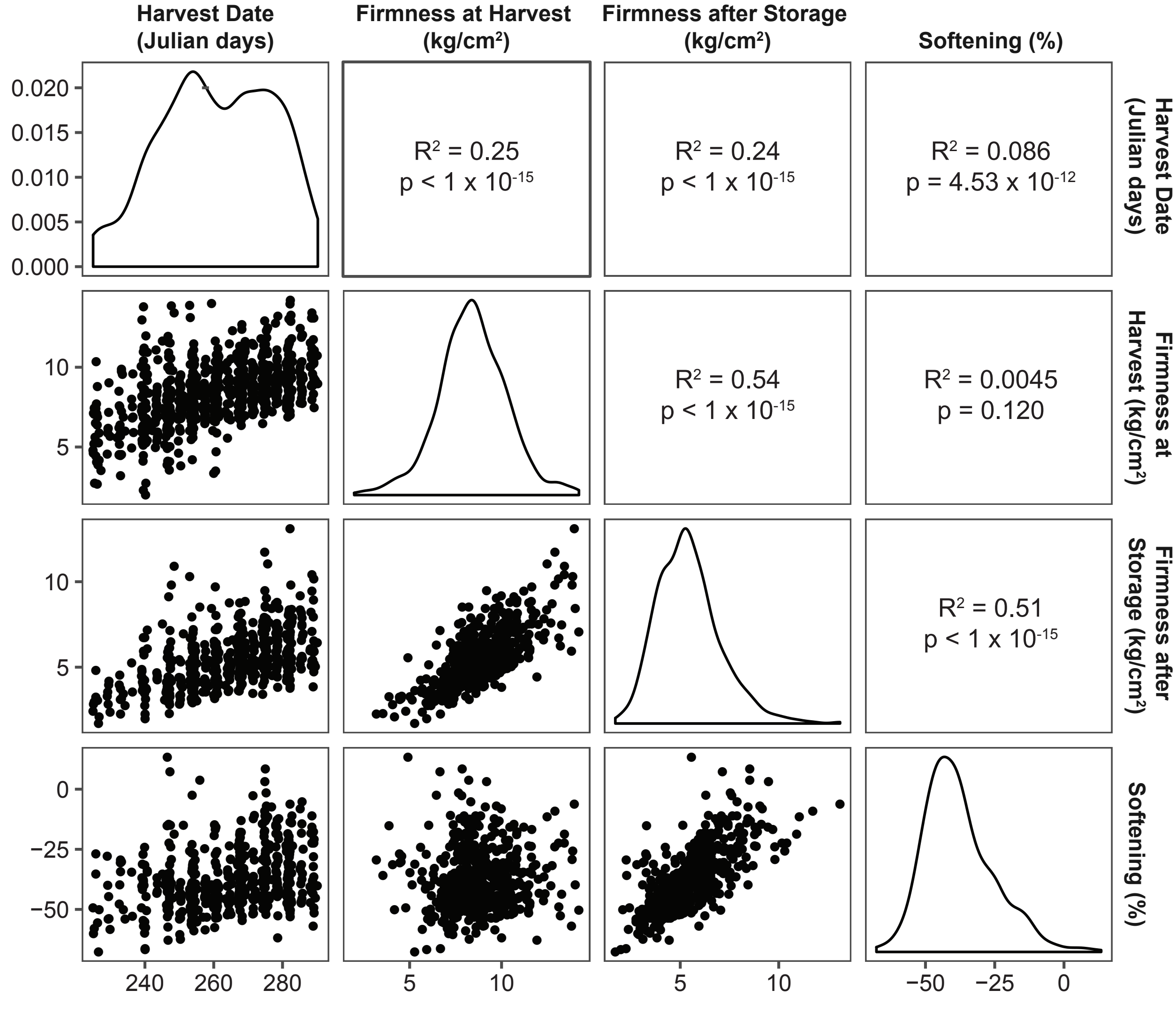

Harvest Date (Julian days)
Firmness at Harvest (kg/cm²)
Firmness after Storage (kg/cm²)
Softening (%)
R² = 0.25
p < 1 x 10-15
R² = 0.24
p < 1 x 10-15
R² = 0.086
p = 4.53 x 10-12
R² = 0.54
p < 1 x 10-15
R² = 0.0045
p = 0.120
R² = 0.51
p < 1 x 10-15
Harvest Date (Julian days)
Firmness at Harvest (kg/cm²)
Firmness after Storage (kg/cm²)
Softening (%)
0.020
0.015
0.010
0.005
0.000
10
5
10
5
0
−25
−50
240
260
280
5
10
5
10
−50
−25
0
