# Supplementary material for: Apple Ripening Is Controlled by a NAC Transcription Factor
Source: Front Genet. 2021 Jun 22;12:671300. doi: 10.3389/fgene.2021.671300 (PMC8258254; doi:10.3389/fgene.2021.671300)
Supplement: Supplementary Figure 1 — Correlations among phenotypes. The distributions of each phenotype are shown as well as dot plots of comparisons between each pair of phenotypes. The results of a Pearson correlation test are provided for each pairwise comparison. [file Data_Sheet_1.zip › Supplementary files/Supp Figures/Image 3.pdf]

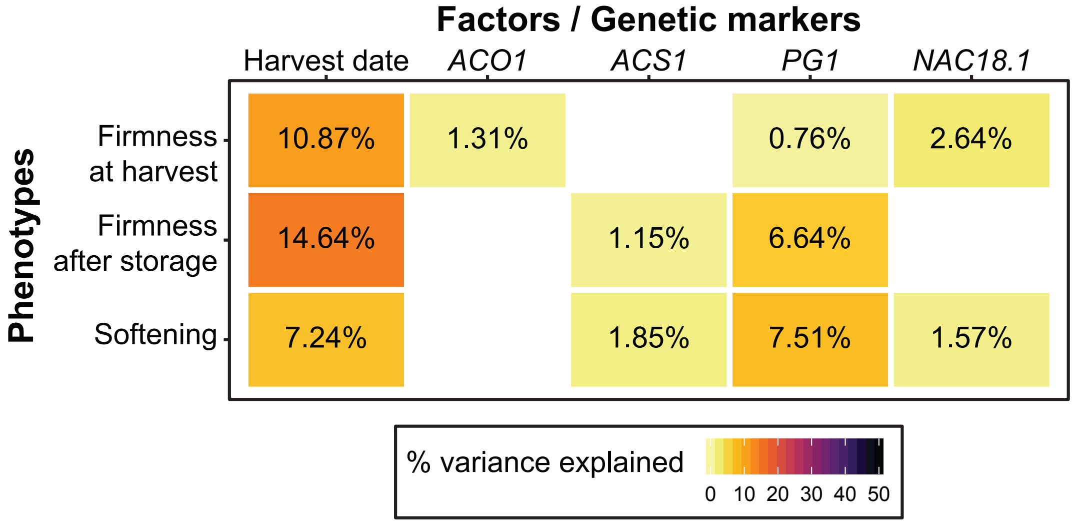
Factors / Genetic markers
Harvest date
ACO1
ACS1
PG1
NAC18.1
Phenotypes
Firmness at harvest
Firmness after storage
Softening
10.87%
1.31%
0.76%
2.64%
14.64%
1.15%
6.64%
7.24%
1.85%
7.51%
1.57%
% variance explained
0
10
20
30
40
50
