# Supplementary figures and images for: Apple Ripening Is Controlled by a NAC Transcription Factor
Source: Front Genet. 2021 Jun 22;12:671300. doi: 10.3389/fgene.2021.671300 (PMC8258254; doi:10.3389/fgene.2021.671300)

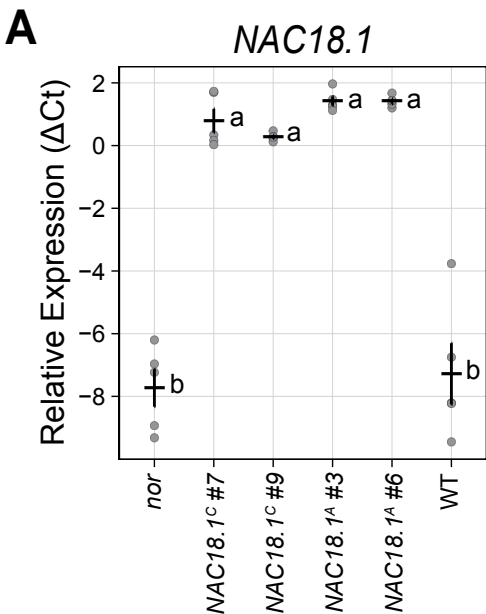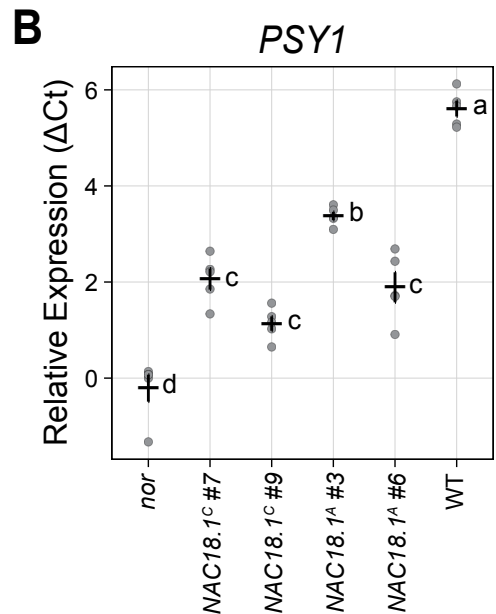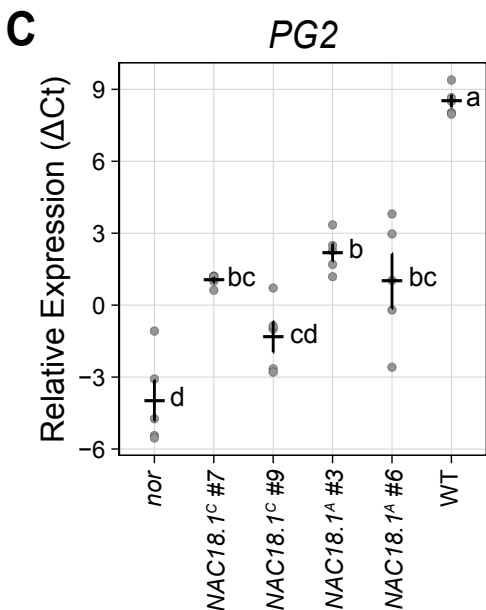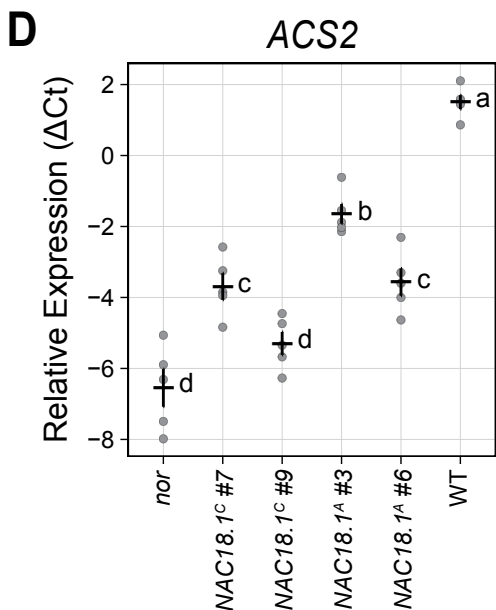

Supplement: Supplementary Figure 1 — Correlations among phenotypes. The distributions of each phenotype are shown as well as dot plots of comparisons between each pair of phenotypes. The results of a Pearson correlation test are provided for each pairwise comparison. [file Data_Sheet_1.zip › Supplementary files/Supp Figures/Image 8.pdf]

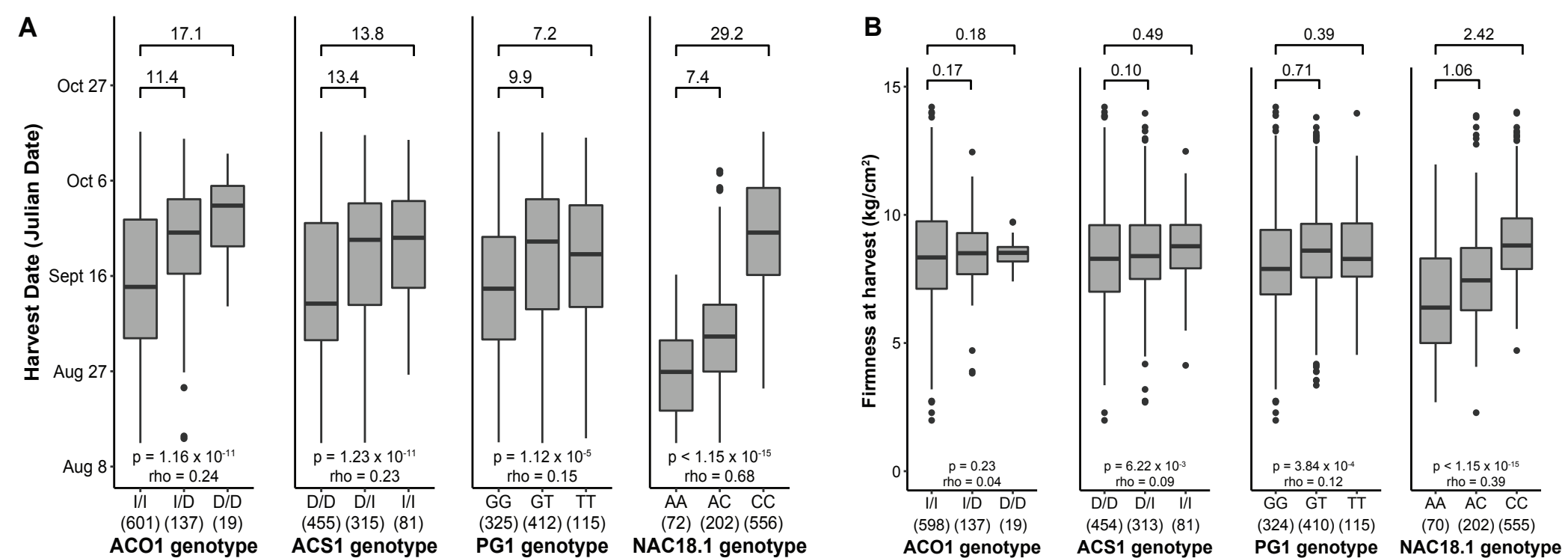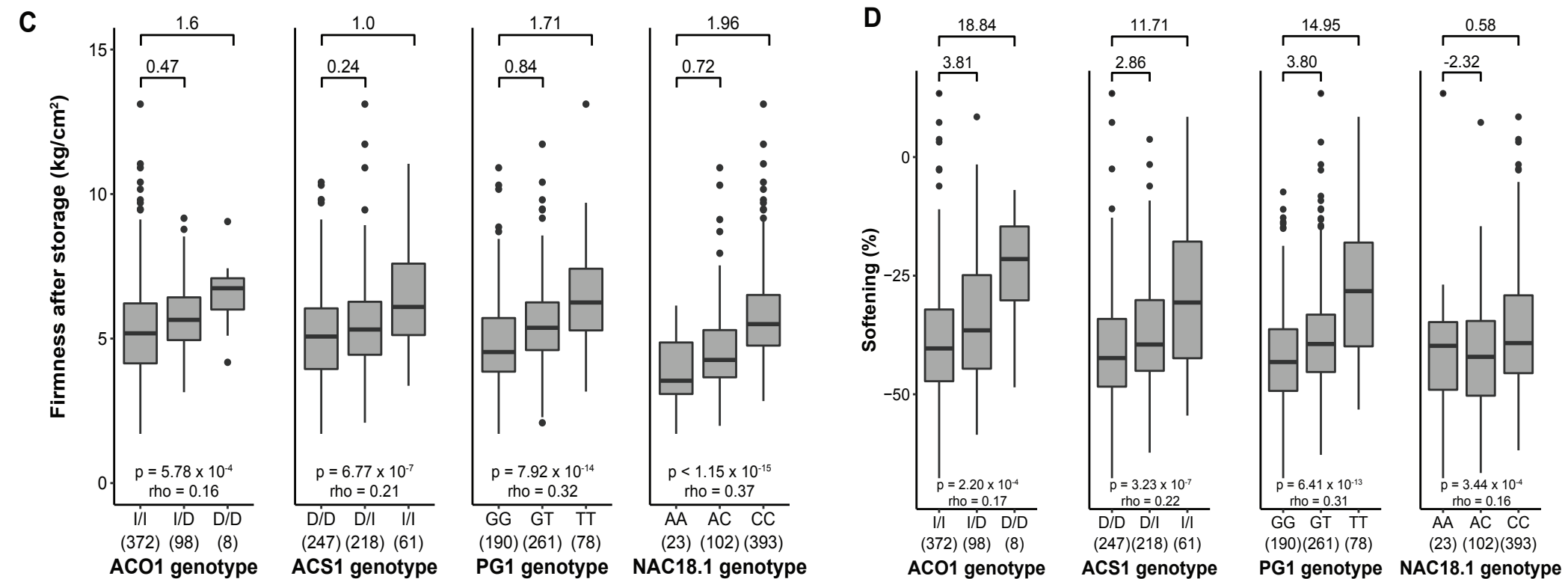

Supplement: Supplementary Figure 1 — Correlations among phenotypes. The distributions of each phenotype are shown as well as dot plots of comparisons between each pair of phenotypes. The results of a Pearson correlation test are provided for each pairwise comparison. [file Data_Sheet_1.zip › Supplementary files/Supp Figures/Image 2.pdf]

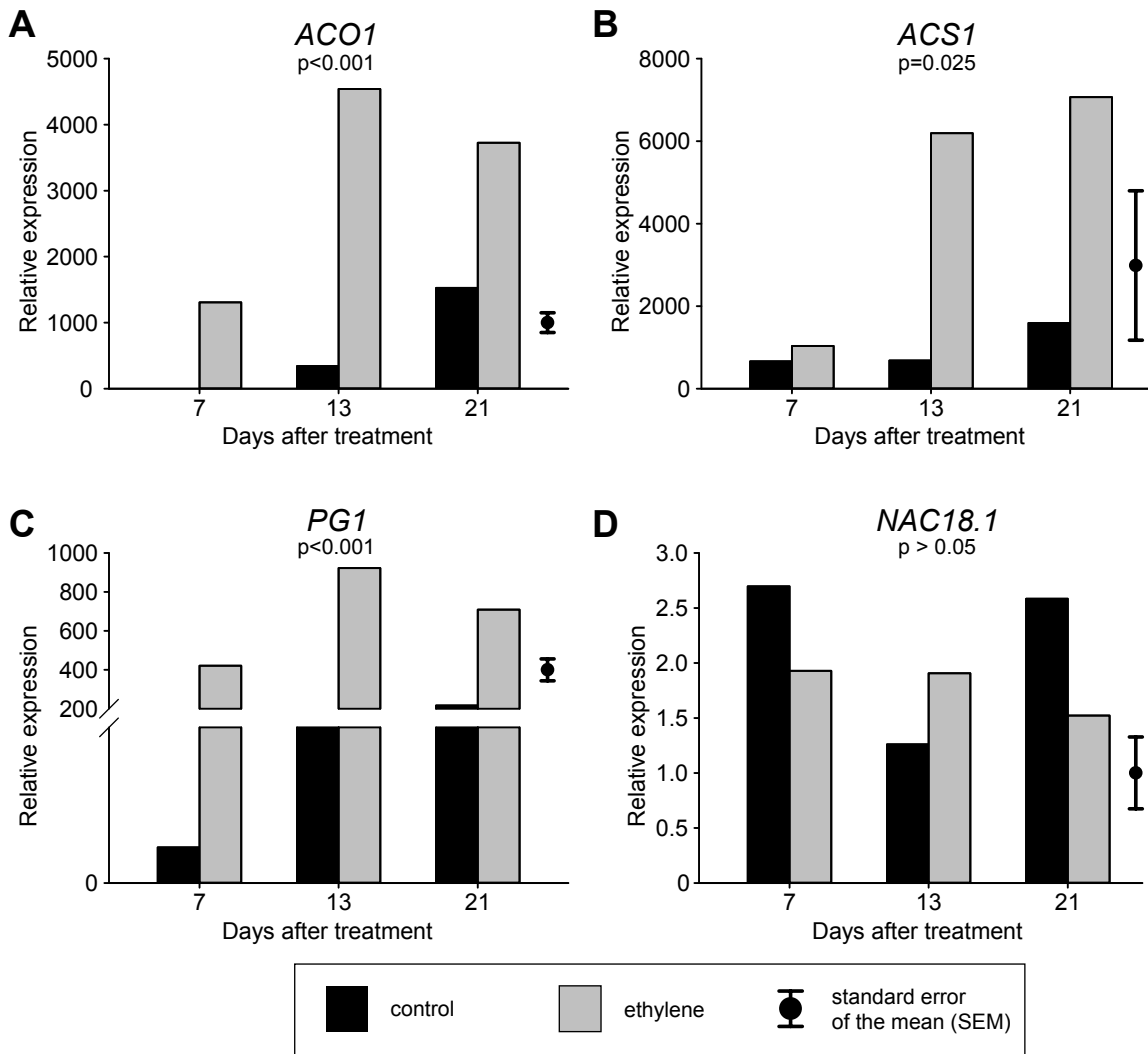

Supplement: Supplementary Figure 1 — Correlations among phenotypes. The distributions of each phenotype are shown as well as dot plots of comparisons between each pair of phenotypes. The results of a Pearson correlation test are provided for each pairwise comparison. [file Data_Sheet_1.zip › Supplementary files/Supp Figures/Image 4.pdf]

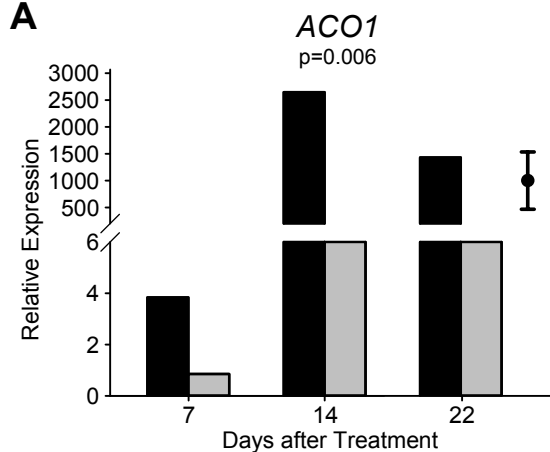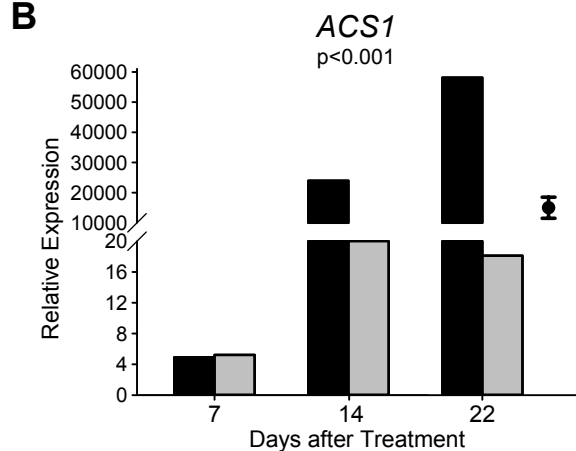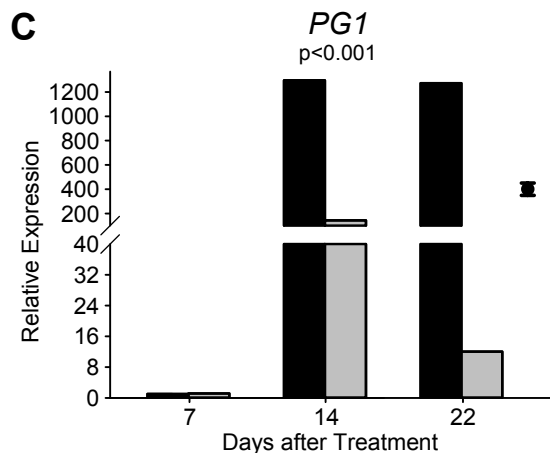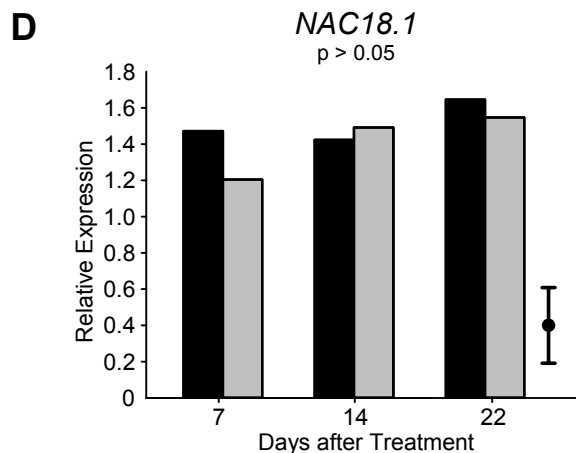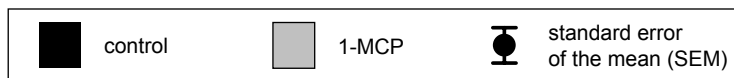

Supplement: Supplementary Figure 1 — Correlations among phenotypes. The distributions of each phenotype are shown as well as dot plots of comparisons between each pair of phenotypes. The results of a Pearson correlation test are provided for each pairwise comparison. [file Data_Sheet_1.zip › Supplementary files/Supp Figures/Image 5.pdf]

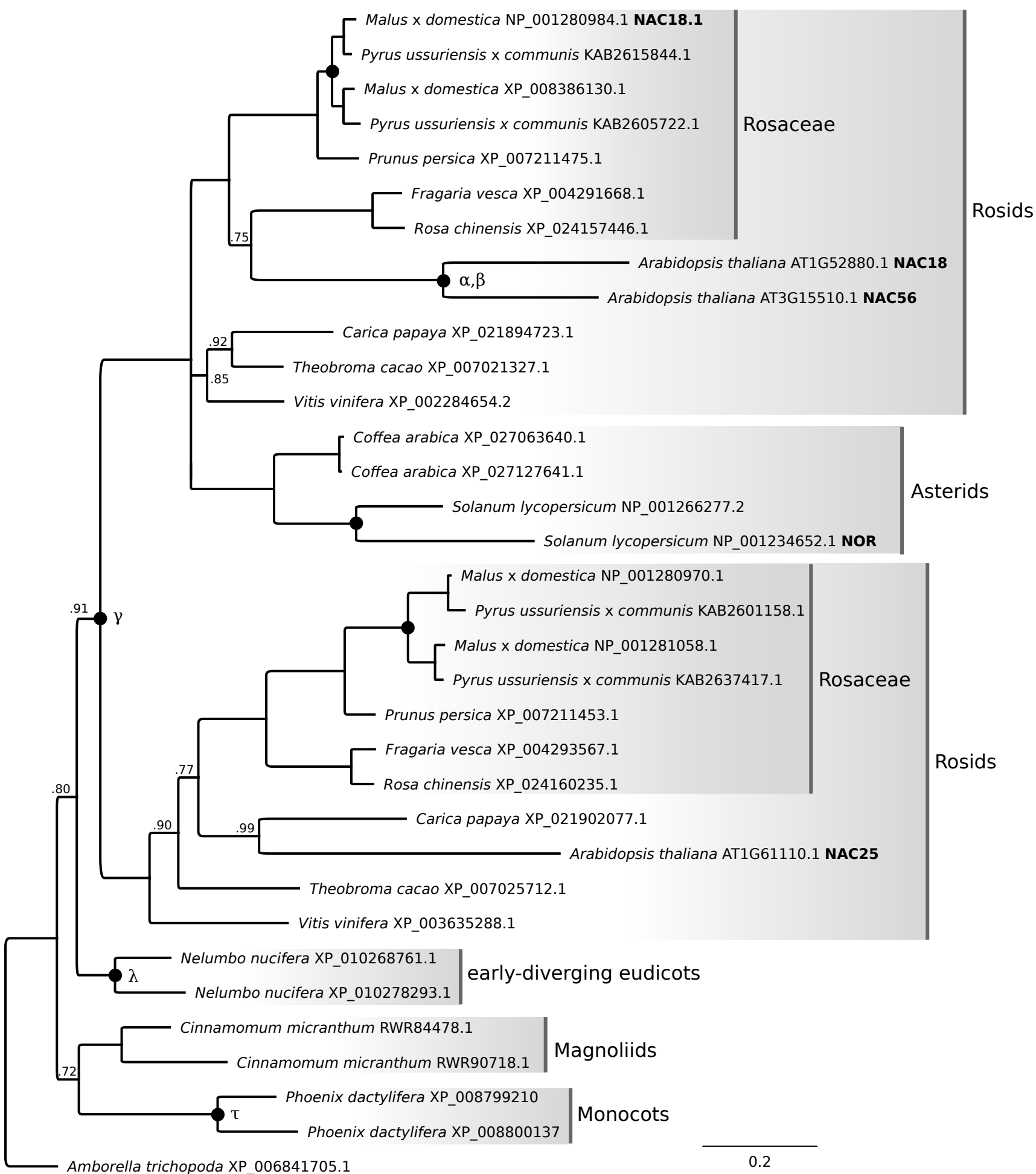

Supplement: Supplementary Figure 1 — Correlations among phenotypes. The distributions of each phenotype are shown as well as dot plots of comparisons between each pair of phenotypes. The results of a Pearson correlation test are provided for each pairwise comparison. [file Data_Sheet_1.zip › Supplementary files/Supp Figures/Image 7.pdf]
