# Supplementary material for: Apple Ripening Is Controlled by a NAC Transcription Factor
Source: Front Genet. 2021 Jun 22;12:671300. doi: 10.3389/fgene.2021.671300 (PMC8258254; doi:10.3389/fgene.2021.671300)
Supplement: Supplementary Figure 1 — Correlations among phenotypes. The distributions of each phenotype are shown as well as dot plots of comparisons between each pair of phenotypes. The results of a Pearson correlation test are provided for each pairwise comparison. [file Image 6.pdf › Supplementary files/Supp Figures/Image 6.pdf]

Anna_2	MESTYSSAGSQQQQHQQPQPQPPPPQPNLPPGFRFHPTDEELVVHYLKKKATAAPLPVAIIAEVDLYKFDPWELPAKATFGEQEWYFFSPRDRKYPNGARPNRAATSGYWKATGTDKPVLTSG
KAZ_95_18-02P-20	MESTYSSAGSQQQQHQQPQPQPPPPQPNLPPGFRFHPTDEELVVHYLKKKATAAPLPVAIIAEVDLYKFDPWELPAKATFGEQEWYFFSPRDRKYPNGARPNRAATSGYWKATGTDKPVLTSG
Petrel_2	MESTYSSAGSQQQQHQQPQPQPPPPQPNLPPGFRFHPTDEELVVHYLKKKATAAPLPVAIIAEVDLYKFDPWELPAKATFGEQEWYFFSPRDRKYPNGARPNRAATSGYWKATGTDKPVLTSG
Early_Harvest	MESTYSSAGSQQQQHQQPQPQPPPPQPNLPPGFRFHPTDEELVVHYLKKKATAAPLPVAIIAEVDLYKFDPWELPAKATFGEQEWYFFSPRDRKYPNGARPNRAATSGYWKATGTDKPVLTSG
Blairmont_2	MESTYSSAGSQQQQHQQPQPQPPPPQPNLPPGFRFHPTDEELVVHYLKKKATAAPLPVAIIAEVDLYKFDPWELPAKATFGEQEWYFFSPRDRKYPNGARPNRAATSGYWKATGTDKPVLTSG
Zestar_2	MESTYSSAGSQQQQHQQPQPQPPPPQPNLPPGFRFHPTDEELVVHYLKKKATAAPLPVAIIAEVDLYKFDPWELPAKATFGEQEWYFFSPRDRKYPNGARPNRAATSGYWKATGTDKPVLTSG
KAZ_96_03-15_2	MESTYSSAGSQQQQHQQPQPQPPPPQPNLPPGFRFHPTDEELVVHYLKKKATAAPLPVAIIAEVDLYKFDPWELPAKATFGEQEWYFFSPRDRKYPNGARPNRAATSGYWKATGTDKPVLTSG
Collins_June	MESTYSSAGSQQQQHQQPQPQPPPPQPNLPPGFRFHPTDEELVVHYLKKKATAAPLPVAIIAEVDLYKFDPWELPAKATFGEQEWYFFSPRDRKYPNGARPNRAATSGYWKATGTDKPVLTSG
Beacon	MESTYSSAGSQQQQHQQPQPQPPPPQPNLPPGFRFHPTDEELVVHYLKKKATAAPLPVAIIAEVDLYKFDPWELPAKATFGEQEWYFFSPRDRKYPNGARPNRAATSGYWKATGTDKPVLTSG
KAZ_96_06-01P-28	MESTYSSAGSQQQQHQQPQPQPPPPQPNLPPGFRFHPTDEELVVHYLKKKATAAPLPVAIIAEVDLYKFDPWELPAKATFGEQEWYFFSPRDRKYPNGARPNRAATSGYWKATGTDKPVLTSG
KAZ_96_07-04	MESTYSSAGSQQQLQQPQPQPPPPQPNLPPGFRFHPTDEELVVHYLKKKATAAPLPVAIIAEVDLYKFDPWELPAKATFGEQEWYFFSPRDRKYPNGARPNRAATSGYWKATGTDKPVLTSG
Directeur_Lesage_2	MESTYSSAGSQQQQHQQPQPQPPPPQPNLPPGFRFHPTDEELVVHYLKKKATAAPLPVAIIAEVDLYKFDPWELPAKATFGEQEWYFFSPRDRKYPNGARPNRAATSGYWKATGTDKPVLTSG
Zestar_1	MECTDSSAGSQQQQHQ----QPPPPQPNLPPGFRFHPTDEELVVHYLKKKATSAPLPVAIIAEVDLYKFDPWELPAKATFGEQEWYFFSPRDRKYPNGARPNRAATSGYWKATGTDKPVLTSG
KAZ_96_03-15_1	MECTDSSAGSQQQQHQ----QPPPPQPNLPPGFRFHPTDEELVVHYLKKKATSAPLPVAIIAEVDLYKFDPWELPAKATFGEQEWYFFSPRDRKYPNGARPNRAATSGYWKATGTDKPVLTSG
Anna_1	MECTDSSAGSQQQQHQ----QPPPPQPNLPPGFRFHPTDEELVVHYLKKKATSAPLPVAIIAEVDLYKFDPWELPAKATFGEQEWYFFSPRDRKYPNGARPNRAATSGYWKATGTDKPVLTSG
Circassian_Apple	MECTDSSAGSQQQQHQ----QPPPPQPNLPPGFRFHPTDEELVVHYLKKKATSAPLPVAIIAEVDLYKFDPWELPAKATFGEQEWYFFSPRDRKYPNGARPNRAATSGYWKATGTDKPVLTSG
Amri	MECTDSSAGSQQQQHQ----QPPPPQPNLPPGFRFHPTDEELVVHYLKKKATSAPLPVAIIAEVDLYKFDPWELPAKATFGEQEWYFFSPRDRKYPNGARPNRAATSGYWKATGTDKPVLTSG
Directeur_Lesage_1	MECTDSSAGSQQQQHQ----QPPPPQPNLPPGFRFHPTDEELVVHYLKKKATSAPLPVAIIAEVDLYKFDPWELPAKATFGEQEWYFFSPRDRKYPNGARPNRAATSGYWKATGTDKPVLTSG
Petrel_1	MECTDSSAGSQQQQHQ----QPPPPQPNLPPGFRFHPTDEELVVHYLKKKATSAPLPVAIIAEVDLYKFDPWELPAKATFGEQEWYFFSPRDRKYPNGARPNRAATSGYWKATGTDKPVLTSG
Coxs_Orange	MECTDSSAGSQQQQHQ----QPPPPQPNLPPGFRFHPTDEELVVHYLKKKATSAPLPVAIIAEVDLYKFDPWELPAKATFGEQEWYFFSPRDRKYPNGARPNRAATSGYWKATGTDKPVLTSG
Deacon_Jones	MECTDSSAGSQQQQHQ----QPPPPQPNLPPGFRFHPTDEELVVHYLKKKATSAPLPVAIIAEVDLYKFDPWELPAKATFGEQEWYFFSPRDRKYPNGARPNRAATSGYWKATGTDKPVLTSG
Golden_Delicious	MECTDSSAGSQQQQHQ----QPPPPQPNLPPGFRFHPTDEELVVHYLKKKATSAPLPVAIIAEVDLYKFDPWELPAKATFGEQEWYFFSPRDRKYPNGARPNRAATSGYWKATGTDKPVLTSG
Blairmont_1	MECTDSSAGSQQQQHQ----QPPPPQPNLPPGFRFHPTDEELVVHYLKKKATSAPLPVAIIAEVDLYKFDPWELPAKATFGEQEWYFFSPRDRKYPNGARPNRAATSGYWKATGTDKPVLTSG
Bouteille	MECTDSSAGSQQQQHQ----QPPPPQPNLPPGFRFHPTDEELVVHYLKKKATSAPLPVAIIAEVDLYKFDPWELPAKATFGEQEWYFFSPRDRKYPNGARPNRAATSGYWKATGTDKPVLTSG

Anna_2	DTQKVGVKKALVFYGGKPPKGMKTNWIMHEYRLADNKPNKKPPGCDLGN-KNSLRLDDWVLCRIYKKNNTHRPMDHEREDSMEDIMGPLMPPSISHVGHHQNMKLHFPKSNSSYGPPFMENDQ
KAZ_95_18-02P-20	DTQKVGVKKALVFYGGKPPKGMKTNWIMHEYRLADNKPNKKPPGCDLGN-KNSLRLDDWVLCRIYKKNNTHRPMDHEREDSMEDIMGPLMPPSISHVGHHQNMKLHFPKSNSSYGPPFMENDQ
Petrel_2	DTQKVGVKKALVFYGGKPPKGMKTNWIMHEYRLADNKPNKKPPGCDLGN-KNSLRLDDWVLCRIYKKNNTHRPMDHEREDSMEDIMGPLMPPSISHVGHHQNMKLHFPKSNSSYGPPFMENDQ
Early_Harvest	DTQKVGVKKALVFYGGKPPKGMKTNWIMHEYRLADNKPNKKPPGCDLGN-KNSLRLDDWVLCRIYKKNNTHRPMDHEREDSMEDIMGPLMPPSISHVGHHQNMKLHFPKSNSSYGPPFMENDQ
Blairmont_2	DTQKVGVKKALVFYGGKPPKGMKTNWIMHEYRLADNKPNKKPPGCDLGN-KNSLRLDDWVLCRIYKKNNTHRPMDHEREDSMEDIMGPLMPPSISHVGHHQNMKLHFPKSNSSYGPPFMENDQ
Zestar_2	DTQKVGVKKALVFYGGKPPKGMKTNWIMHEYRLADNKPNKKPPGCDLGN-KNSLRLDDWVLCRIYKKNNTHRPMDHEREDSMEDIMGPLMPPSISHVGHHQNMKLHFPKSNSSYGPPFMENDQ
KAZ_96_03-15_2	DTQKVGVKKALVFYGGKPPKGMKTNWIMHEYRLADNKPNKKPPGCDLGN-KNSLRLDDWVLCRIYKKNNTHRPMDHEREDSMEDIMGPLMPPSISHVGHHQNMKLHFPKSNSSYGPPFMENDQ
Collins_June	DTQKVGVKKALVFYGGKPPKGMKTNWIMHEYRLADNKPNKKPPGCDLGN-KNSLRLDDWVLCRIYKKNNTHRPMDHEREDSMEDIMGPLMPPSISHVGHHQNMKLHFPKSNSSYGPPFMENDQ
Beacon	DTQKVGVKKALVFYGGKPPKGMKTNWIMHEYRLADNKPNKKPPGCDLGN-KNSLRLDDWVLCRIYKKNNTHRPMDHEREDSMEDIMGPLMPPSISHVGHHQNMKLHFPKSNSSYGPPFMENDQ
KAZ_96_06-01P-28	DTQKVGVKKALVFYGGKPPKGMKTNWIMHEYRLADNKPNKKPPGCDLGN-KNSLRLDDWVLCRIYKKNNTHRPMDHEREDSMEDIMGPLMPPSISHVGHHQNMKLHFPKSNSSYGPPFMENDQ
KAZ_96_07-04	DTQKVGVKKALVFYGGKPPKGMKTNWIMHEYRLADNKPNKKPPGCDLGN-KNSLRLDDWVLCRIYKKNNTHRPMDHEREDSMEDIMGPLMPPSISHVGHHQNMKLHFPKSNSSYGPPFMENDQ
Directeur_Lesage_2	DTQKVGVKKALVFYGGKPPKGMKTNWIMHEYRLADNKPNKKPPGCDLGN-KNSLRLDDWVLCRIYKKNNTHRPMDHEREDSMEDIMGPLMPPSISHVGHHQNMKLHFPKSNSSYGPPFMENDQ
Zestar_1	DTQKVGVKKALVFYGGKPPKGIKTNWIMHEYRLADNKPNNKPPGCDLGNKKNSLRLDDWVLCRIYKKNNTHRPMDHEREDSMEDIMGPLMPPSISHVGHHQNMKLHFPKSNSSYGPPFMENDQ
KAZ_96_03-15_1	DTQKVGVKKALVFYGGKPPKGIKTNWIMHEYRLADNKPNNKPPGCDLGNKKNSLRLDDWVLCRIYKKNNTHRPMDHEREDSMEDIMGPLMPPSISHVGHHQNMKLHFPKSNSSYGPPIMENDQ
Anna_1	DTQKVGVKKALVFYGGKPPKGIKTNWIMHEYRLADNKPNNKPPGCDLGNKKNSMRLDDWVLCRIYKKNNTHRPMDHEREDSMEDIMGPLMSPSISHVGHHQNMKLHFPKSNSSYGPPFMENDQ
Circassian_Apple	DTQKVGVKKALVFYGGKPPKGIKTNWIMHEYRLADNKPNNKPPGCDLGNKKNSLRLDDWVLCRIYKKNNTHRPMDHEREDSMEDIMGPLMPPSISHVGHHQNMKLHFPKSNSSYGPPIMENDQ
Amri	DTQKVGVKKALVFYGGKPPKGIKTNWIMHEYRLADNKPNNKPPGCDLGNKKNSLRLDDWVLCRIYKKNNTHRPMDHEREDSMEDIMGPLMPPSISHVGHHQNMKLHFPKSNSSYGPPIMENDQ
Directeur_Lesage_1	DTQKVGVKKALVFYGGKPPKGIKTNWIMHEYRLADNKPNNKPPGCDLGNKKNSLRLDDWVLCRIYKKNNTHRPMDHEREDSMEDIMGPLMPPSISHVGHHQNMKLHFPKSNSSYGPPIMENDQ
Petrel_1	DTQKVGVKKALVFYGGKPPKGIKTNWIMHEYRLADNKPNNKPPGCDLGNKKNSLRLDDWVLCRIYKKNNTHRPMDHEREDSMEDIMGPLMPPSISHVGHHQNMKLHFPKSNSSYGPPIMENDQ
Coxs_Orange	DTQKVGVKKALVFYGGKPPKGIKTNWIMHEYRLADNKPNNKPPGCDLGNKKNSLRLDDWVLCRIYKKNNTHRPMDHEREDSMEDIMGPLMPPSISHVGHHQNMKLHFPKSNSSYGPPFMENDQ
Deacon_Jones	DTQKVGVKKALVFYGGKPPKGIKTNWIMHEYRLADNKPNNKPPGCDLGNKKNSLRLDDWVLCRIYKKNNTHRPMDHEREDSMEDIMGPLMPPSISHVGHHQNMKLHFPKSNSSYGPPFMENDQ
Golden_Delicious	DTQKVGVKKALVFYGGKPPKGIKTNWIMHEYRLADNKPNNKPPGCDLGNKKNSMRLDDWVLCRIYKKNNTHRPMDHEREDSMEDIMGPLMPPSISHVGHHQNMKLHFPKSNSSYGPPFMENDQ
Blairmont_1	DTQKVGVKKALVFYGGKPPKGIKTNWIMHEYRLADNKPNNKPPGCDLGNKKNSMRLDDWVLCRIYKKNNTHRPMDHEREDSMEDIMGPLMPPSISHVGHHQNMKLHFPKSNSSYGPPFMENDQ
Bouteille	DTQKVGVKKALVFYGGKPPKGIKTNWIMHEYRLADNKPNNKPPGCDLGNKKNSMRLDDWVLCRIYKKNNTHRPMDHEREDSMEDIMGPLMPPSISHVGHHQNMKLHFPKSNSSYGPPFMENDQ

Anna_2	IFFDGIMSSTDGSASTHQLQLKRPIVPSWYWNNQEDDQTAAVPSSSKRIQLHQLDNGTNSNGDGINGGSTNNNSCSIANLLSELPETPPLHQHAVLGSLGDGIFQTPYQLPGKNWYSESNLG
KAZ_95_18-02P-20	IFFDGIMSSTDGSASTHQLQLKRPIVPSWYWNNQEDDQTAAVPSSSKRIQLHQLDNGTNSNGDGINGGSTNNNSCSIANLLSELPETPPLHQHAVLGSLGDGIFQTPYQLPGKNWYSESNLG
Petrel_2	IFFDGIMSSTDGSASTHQLQLKRPIVPSWYWNNQEDDQTAAVPSSSKRIQLHQLDNGTNSNGDGINGGSTNNNSCSIANLLSELPETPPLHQHAVLGSLGDGIFQTPYQLPGKNWYSESNLG
Early_Harvest	IFFDGIMSSTDGSASTHQLQLKRPIVPSWYWNNQEDDQTAAVPSSSKRIQLHQLDNGTNSNGDGINGGSTNNNSCSIANLLSELPETPPLHQHAVLGSLGDGIFQTPYQLPGKNWYSESNLG
Blairmont_2	IFFDGIMSSTDGSASTHQLQLKRPIVPSWYWNNQEDDQTAAVPSSSKRIQLHQLDNGTNSNGDGINGGSTNNNSCSIANLLSELPETPPLHQHAVLGSLGDGIFQTPYQLPGKNWYSESNLG
Zestar_2	IFFDGIMSSTDGSASTHQLQLKRPIVPSWYWNNQEDDQTAAVPSSSKRIQLHQLDNGTNSNGDGINGGSTNNNSCSIANLLSELPETPPLHQHAVLGSLGDGIFQTPYQLPGKNWYSESNLG
KAZ_96_03-15_2	IFFDGIMSSTDGSASTHQLQLKRPIVPSWYWNNQEDDQTAAVPSSSKRIQLHQLDNGTNSNGDGINGGSTNNNSCSIANLLSELPETPPLHQHAVLGSLGDGIFQTPYQLPGKNWYSESNLG
Collins_June	IFFDGIMSSTDGSASTHQLQLKRPIVPSWYWNNQEDDQTAAVPSSSKRIQLHQLDNGTNSNGDGINGGSTNNNSCSIANLLSELPETPPLHQHAVLGSLGDGIFQTPYQLPGKNWYSESNLG
Beacon	IFFDGIMSSTDGSASTHQLQLKRPIVPSWYWNNQEDDQTAAVPSSSKRIQLHQLDNGTNSNGDGINGGSTNNNSCSIANLLSELPETPPLHQHAVLGSLGDGIFQTPYQLPGKNWYSESNLG
KAZ_96_06-01P-28	IFFDGIMSSTDGSASTHQLQLKRPIVPSWYWNNQEDDQTAAVPSSSKRIQLHQLDNGTNSNGDGINGGSTNNNSCSIANLLSELPETPPLHQHAVLGSLGDGIFQTPYQLPGKNWYSESNLG
KAZ_96_07-04	IFFDGIMSSTDGSASTHQLQLKRPIVPSWYWNNQEDDQTAAVPSSSKRIQLHQLDNGTNSNGDGINGGSTNNNSCSIANLLSELPETPPLHQHAVLGSLGDGIFQTPYQLPGKNWYSESNLG
Directeur_Lesage_2	IFFDGIMSSTDGSASTHQLQLKRPIVPSWYWNNQEDDQTAAVPSSSKRIQLHQLDNGTNSNGDGINGGSTNNNSSSIANLLSQLPQTPPLHQHAVLGSLGDGIFRTPYQLPGMNWYSESNLG
Zestar_1	IFFDGIMSSTDGSASTHQLQLKRPIVPSWYWNNQEDDQTAAVPSSSKRIQLHQLDNGTNSNGDGINGGSTNNNSCSIANLLSELPETPPLHQHAVLGSLGDGIFQTPYQLPGKNWYSESNLG
KAZ_96_03-15_1	IFFDGIMSSTDGSASTHQLQLKRPIVPSWYWNNQEDDQTAAVPSSSKRIQLHQLDNGTNSNGDGINGGSTNNNSCSIANLLSELPETPPLHQHAVLGSLGDGIFQTPYQLPGKNWYSESNLG
Anna_1	IFFDGIMSSTDGSASTHQLQLKRPIVPSWYWNNQEDDQTAADPSSSKRIQLHQLDNGTNSNGDGINGGSTNNNSCSIANLLSELPETPPLHQHAVLGSLGDGIFQTPYQLPGKNWYSESNLG
Circassian_Apple	IFFDGIMSSTDGSASTHQLQLKRPIVPSWYWNNQEDDQTAADPSSSKRIQLHQLDNGTNSNGAGINGGSTNNNSSSIANLLSQLPQTPPLHQHAVLGSLGDGIFRTPYQLPGMNWYSESNLG
Amri	IFFDGIMSSTDGSASTHQLQLKRPIVPSWYWNNQEDDQTAADPSSSKRIQLHQLDNGTNSNGAGINGGSTNNNSSSIANLLSQLPQTPPLHQHAVLGSLGDGIFRTPYQLPGMNWYSESNLG
Directeur_Lesage_1	IFFDGIMSSTDGSASTHQLQLKRPIVPSWYWNNQEDDQTAADPSSSKRIQLHQLDNGTNSNGAGINGGSTNNNSSSIANLLSQLPQTPPLHQHAVLGSLGDGIFRTPYQLPGMNWYSESNLG
Petrel_1	IFFDGIMSSTDGSASTHQLQLKRPIVPSWYWNNQEDDQTAADPSSSKRIQLHQLDNGTNSNGAGINGGSTNNNSSSIANLLSQLPQTPPLHQHAVLGSLGDGIFRTPYQLPGMNWYSESNLG
Coxs_Orange	IFFDGIMSSTDGSASTHQLQLKRPIVPSWYWNNQEDDQTAADPSSSKRIQLHQLDNGTNSNGAGINGGSTNNNSSSIANLLSQLPQTPPLHQHAVLGSLGDGIFRTPYQLPGMNWYSESNLG
Deacon_Jones	IFFDGIMSSTDGSASTHQLQLKRPIVPSWYWNNQEDDQTAADPSSSKRIQLHQLDNGTNSNGAGINGGSTNNNSSSIANLLSQLPQTPPLHQHAVLGSLGDGIFRTPYQLPGMNWYSESNLG
Golden_Delicious	IFFDGIMSSTDGSASTHQLQLKRPIVPSWYWNNQEDDQTAADPSSSKRIQLHQLDNGTNSNGAGINGGSTNNNSSSIANLLSQLPQTPPLHQHAVLGSLGDGIFRTPYQLPGMNWYSESNLG
Blairmont_1	IFFDGIMSSTDGSASTHQLQLKRPIVPSWYWNNQEDDQTAADPSSSKRIQLHQLDNGTNSNGAGINGGSTNNNSSSIANLLSQLPQTPPLHQHAVLGSLGDGIFRTPYQLPGMNWYSESNLG
Bouteille	IFFDGIMSSTDGSASTHQLQLKRPIVPSWYWNNQEDDQTAADPSSSKRIQLHQLDNGTNSNGAGINGGSTNNNSSSIANLLSQLPQTPPLHQHAVLGSLGDGIFRTPYQLPGMNWYSESNLG
